# Calendar

**DOI:** 10.1155/S1463924688000148

**Published:** 1988


					Journal of Automatic Chemistry, Vol. 10, No. 2 (April-June 1988), p. 66
Calendar
18-19 May 1988 Laboratory Manchester: New Century Hall.,
Manchester, UK. Contact Curtis Steadman & Partners
Ltd, Saffron Walden (as above).
1988
APRIL
13-14 April 1988 Laboratory Science and Technology Show:
Kelsey Kerridge Sports Hall, Cambridge, UK. Contact Curtis
Steadman & Partners Ltd, The Hub, Emson Close,
Saffron Walden, Essex CB 10 1HL, UK.
17-20 April 1988 International Conference on Flow-Injection
Analysis: Las Vegas, Nevada, USA. Contact Dr G. E. Pacey,
Flow Analysis IV, Department of Chemistry, Miami
University, Oxford, OH 45056, USA.
19-22 April 1988 Analytica 88- llth International Exhibition
with International Conference: Munich, FR Germany. Contact.
Mfinchener Messe und Ausstellungsgesellschaft GmbH,
Postfach 121009, D8000 Munich I, FR Germany.
20-21 April 1988 Rheology Training Course: The Crest Hotel,
Luton, UK. Contact Miss M. Fearon, RTI Ltd, 120-122
Woodgrange Road, Forest Gate, London E7 0EW.
27-29 April 1988 Symposium on Handling ofEnvironmental
and Biological Samples in Chromatography: Sandoz Auditorium,
Basel, Switzerland. Contact M. Frei-Hiusler, IAEAC,
Postfach 46, 4123 Allschwil 2, Switzerland.
JUNE
5-11 June 1988 Achema 88- Chemical Engineering Exhibition
& Congress: Frankfurt, FR Germany. Contact Dechema,
Postfach 970146, D6000 Frankfurt/Main 97, FR Ger-
many.
SEPTEMBER
5-9 September 1988 Analytical Plasma Spectrometry: Lough-
borough, UK. Contact Mrs J. Stirling, Department of
Chemistry, Loughborough University of Technology,
Loughborough, Leicestershire LEll 3TU, UK.
12 September 1988 Atomic Absorption Spectrometry: Lough-
borough, UK. Contact Mrs J. Stirling (as above).
14-16 September 1988 Modern Electroanalytical Methods:
Loughborough, UK. Contact Mrs J. Stirling (as above).
19-23 September 1988 Microcomputers in the Laboratory:
Loughborough, UK. Contact Mrs J. Stirling (as above).
MAY
2-6 May 1988 Het Instrument 88: Utrecht, The Netherlands.
Contact Het Instrument, PO Box 152, 3760 AD Soest,
The Netherlands.
OCTOBER
11-13 October 1988 The British Laboratory Week: Grand
Hall, Olympia, London. Contact Curtis Steadman &
Partners, Saffron Walden (as above).
5 May 1988 Laboratory Information Management Systems:
Welwyn Garden City, UK. Contact the Analytical Division,
Royal Society of Chemistry, Burlington House, London
W1V 0BN.
10 May 1988 Chemiluminescence and Fluorescence in Applied
Biosciences: London. Contact Mr S. Langer, Royal Society
of Chemistry, Burlington House, London W1V 0BN.
12-13 May 1988 International Congress on Polyurethanes:
Milan, Italy. Contact Congress Secretariat, Assocoma-
plast, Centro Commerciale Milanofiori, Palazzo F2,
20090 Assago, Milano, Italy.
16-18 May 1988 Thermal Analysis in Research and Production
-an intensive short course: Basel, Switzerland. Contact
Professor Edith A. Turi, Polytechnic University, 5
Oxford Drive, Livingston, New Jersey 07039, USA.
NOVEMBER
9-12 November 1988 Clinic&Lab World Trade
Centre, Singapore. Contact Curtis Steadman & Partners,
Saffron Walden (as above).
1989
25-28 September 1989 Third International Symposium on
Kinetics in Analytical Chemistry: Dubrovnik, Yugoslavia. Con-
tact Professor Gordana A. Milonovi:, Department of
Chemistry, University of Belgrade, KAC PO Box 550,
11001 Belgrade, Yugoslavia.
66

				

